# When Stress Predicts a Shrinking Gene Pool, Trading Early Reproduction for Longevity Can Increase Fitness, Even with Lower Fecundity

**DOI:** 10.1371/journal.pone.0006055

**Published:** 2009-06-25

**Authors:** William C. Ratcliff, Peter Hawthorne, Michael Travisano, R. Ford Denison

**Affiliations:** Ecology Evolution and Behavior, University of Minnesota, St. Paul, Minnesota, United States of America; University of Leeds, United Kingdom

## Abstract

**Background:**

Stresses like dietary restriction or various toxins increase lifespan in taxa as diverse as yeast, *Caenorhabditis elegans*, *Drosophila* and rats, by triggering physiological responses that also tend to delay reproduction. Food odors can reverse the effects of dietary restriction, showing that key mechanisms respond to information, not just resources. Such environmental cues can predict population trends, not just individual prospects for survival and reproduction. When population size is increasing, each offspring produced earlier makes a larger proportional contribution to the gene pool, but the reverse is true when population size is declining.

**Principal Findings:**

We show mathematically that natural selection can favor facultative delay in reproduction when environmental cues predict a decrease in total population size, even if lifetime fecundity decreases with delay. We also show that increased reproduction from waiting for better conditions does not increase fitness (proportional representation) when the whole population benefits similarly.

**Conclusions:**

We conclude that the beneficial effects of stress on longevity (hormesis) in diverse taxa are a side-effect of delaying reproduction in response to environmental cues that population size is likely to decrease. The reversal by food odors of the effects of dietary restriction can be explained as a response to information that population size is less likely to decrease, reducing the chance that delaying reproduction will increase fitness.

## Introduction

Food odors can reverse the life-extending effects of dietary restriction [1]. Intermittent fasting increases longevity in vertebrates and *Caenorhabditis elegans*, even when there is little to no reduction in overall calorie consumption [2], [3]. A crowding pheromone delays reproduction and extends lifespan in *C. elegans* [4], [5]. Water that contained starving pond snails induces delay in pond snail egg development, doubling embryo maturation time [6]. Repeated mild heat stress extends lifespan in *Drosophila* at the expense of fecundity [7]. Cold stress induces diapause in *Drosophila*, halting reproduction and extending lifespan [8]. Low doses of many plant-defense toxins, including some that are not antioxidants, can extend lifespan [9]. We show that phenomena like these can be explained as responses of reproductive timing to information that predicts, not an individual's own particular prospects, but rather changes in overall population size.

The evolution of reproductive delay has previously been explained by three main hypotheses. First, reproductive delay can increase fitness when older individuals are more reproductively successful than they would have been earlier [10]–[12], even if fecundity with delay is less than a younger individual might have achieved under better conditions [13]. Second, reproductive delay can act as a bet-hedging mechanism, increasing fitness in unpredictable environments [11], [14]. The third hypothesis, by far the most widely-cited in the aging literature, is that reproductive delay during periods of adversity promotes survival until conditions improve, thereby increasing individual fecundity and fitness [2], [4], [8], [15]–[21]. We show that delaying reproduction can be adaptive even when none of the above hypotheses are true. Our alternative hypothesis considers the fitness consequences of plasticity in reproductive timing in response to environmental cues predicting changes in overall population size.

Changes in population size play a fundamental role in determining the evolutionary consequences of the timing of reproduction [22], [23]. Stable population size favors early reproduction, because of the risk of dying before the next opportunity to reproduce. In growing populations, early reproduction is favored even more strongly, because each offspring added to a smaller current gene pool is a larger proportional contribution than one added to a larger future gene pool [22], [24]. Conversely, natural selection favors delayed reproduction when overall population size is decreasing [22], [25]. Evolution of reproductive timing in shrinking populations has often been considered “somewhat academic” [26] because “a population with a negative growth rate would soon go extinct” [27]. However, short-term fluctuations in population size are common. Here, we show that facultative delay in reproduction during periodic population declines enhances fitness. A key point is that, if organisms can use environmental cues to predict population decline and consequentially delay reproduction, fitness can be enhanced even without increases in lifetime fecundity.

## Results

There are two kinds of cues relevant to delaying reproduction: those specific to individuals and those that apply to the population as a whole. As an example, dietary restriction may directly affect individuals, and may also provide information about future survival and reproduction of an individual or the population as a whole. We will start with some simplifying assumptions before considering more general cases.

Consider a semelparous species with haploid genetics and no parental care. Each individual reproduces only once, at either one or two years of age, then dies. Assume that all reproduction occurs in summer and juveniles (or adults delaying reproduction) die only in winter.

An individual increases its lifetime fecundity by delaying reproduction only if
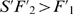
(1)where *S*′ is the focal individual's chance of surviving to reproduce in year 2 if it delays reproduction, and *F_1_′* and *F_2_′* are its expected fecundity as a one- or two-year-old. Given the trade-off between current and future reproduction – semelparity is an extreme example – delaying reproduction might increase fitness if 1) fecundity increases with age or experience, or 2) fecundity will increase due to improvement in conditions (e.g., weather or food). To emphasize our main point, we initially assume that neither is true. This would be the case if there is no benefit to age and if favorable and unfavorable periods last long enough, relative to the generation time, that an individual cannot wait until conditions change to reproduce. We therefore assume no difference between years 1 and 2, so *F_2_′* = *F_1_′*. Because *S′* cannot exceed one, delaying reproduction cannot increase expected lifetime fecundity.

While sometimes used synonymously, lifetime fecundity is not the same as fitness. Natural selection depends, not on absolute numbers, but on proportional representation in the population [22], [26]. If we calculate changes in proportional representation immediately after year-2 reproduction, then a rare allele for facultative delay in reproduction will increase in frequency, within a population of first-year reproducers, if and only if
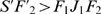
(2)where *F_1_* and *F_2_* are year-1 and year-2 fecundity for the overall population and *J_1_* is the fraction of those juveniles born in year 1 that survive to reproduce in year 2. In terms of the information available to the focal individual in year 1 that is relevant to delaying reproduction, we assume that carry-over effects of year-1 individual condition (fat reserves, etc.) to its year-2 fecundity *F_2_′* are negligible, relative to shared-environment effects on year-2 fecundity of the whole population. Therefore *F_2_′* = *F_2_*. Delaying reproduction is then favored if
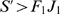
(3)The left side of Eqn. 3 is the focal-individual-specific chance of adult survival to year 2, while the right side is overall population change, the ratio of total population in year 2 to that in year 1. If *S′* takes its maximum possible value of 1.0, then delaying reproduction increases the focal individual's fitness (proportional representation in the population) if and only if population decreases from year 1 to year 2. Even if adult survival is uncertain, a more drastic population decrease can still favor delaying reproduction. For example, natural selection will favor facultative delay in reproduction if there is reliable information that the population will decrease by 50% (*F_1_J_1_* = 0.5) and the individual-specific chance of adult survival *S′* is >50%. Note that Eqn. 3 does not include *J_2_*, so the benefits of delay also do not depend on whether juvenile survival is better in year 2 than in year 1.

The above analysis assumed that the duration of favorable or unfavorable conditions greatly exceeds individual lifespan and that older individuals receive no benefit from growth or experience, so that reproductive delay never increases individual fecundity. In real populations, however, such benefits may be common. Relaxing both assumptions, we show that a genotype with facultative delay in reproduction in response to cues predicting population decline can invade a population of first-year reproducers and that facultative delay is an evolutionarily stable strategy (ESS). Further, we show that the increase in reproductive success that individuals gain by delaying reproduction until conditions improve does not necessarily increase their relative fitness.

Consider a semelparous population composed of a genotype (A) that reproduces at age one year (like an annual plant) and another genotype (FD) that facultatively delays reproduction for one year when conditions are bad (i.e., if population is likely to decrease), then reproduces the next year regardless of year quality (like some facultative biennials). Because no individual lives more than two years, we can enumerate all possible fitness effects of variation among years by considering four possible two-year combinations of good and bad year quality: BB, GG, GB, and BG. An initially rare FD can invade a population of A when the two-year growth rate (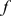, the ratio of individuals in spring of year 3 to those in spring of year 1) of FD is greater than the overall population growth rate, i.e., that of A. Let *F* be average adult fecundity, *J* be the average probability that a juvenile will survive to reproduce as an adult in the next year, *δ* (constrained so that *δ>FJ-1*) be the difference in *FJ* between an average year and a good or bad year, *S* be the probability that an adult delaying reproduction during a bad year survives to reproduce the next year, and α be the reproductive advantage of second year adults. All other conditions are the same as in the first model.

During two successive good years (GG) both genotypes reproduce each year. Growth rates are then:
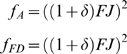
(4)In an expanding population 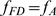 and so FD cannot invade a population of A.

Growth rates during two bad years (BB):
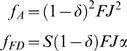
(5)Here 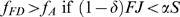, meaning that FD can invade if overall population decline in a bad year is worse than the consequences of delay: increased reproduction as a two-year old (if α>1) but a decreased probability of surviving to reproduce.

Growth rates when a good year follows a bad year (BG):
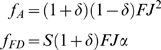
(6)Again, we find that 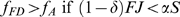.

Finally, when a bad year follows a good year (GB), both genotypes reproduce the first year, and the FD delays reproduction during the second. As before, we compare expected genotype growth rates across two years, but because the fitness consequence of FD's delay during the second year depends on year 3 quality, we calculated the expected value for 3^rd^ year reproduction assuming G and B occur with equal probability:
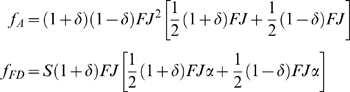
(7)As with BG and BB, we find that 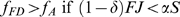.

In short, neither genotype gains an advantage during a population expansion (Eq. 4), but a rare FD can invade a population of A in any series of years involving population decline (Eq. 5–7).

Can FD maintain dominance once common? FD is an ESS if A cannot invade when rare. We find that 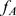 is not greater than 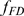 during population expansion (Eq. 4), and so cannot invade, and that 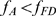 during a series of years that include population decline when 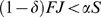. Thus, FD is an ESS under the same conditions that it can invade a population of genotype A.

Because the success of the facultative-delay strategist FD depends on parameter values, we found the critical values under which FD dominates A, specifically focusing on the probability (*S*) that a 1-year old delaying reproduction survives to reproduce in year 2. Holding *α*, *FJ*, and *δ* constant, the minimum value for *S* required for FD to obtain a relative fitness advantage is:
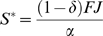
(8)Qualitatively, Eqn. 8 shows that facultative delay in reproduction increases fitness when survival from year 1 to 2 (*S*) is large or when either survival from birth to year 1 (*J*) or fecundity (*F*) is small, so that population decreases. Large variation among years (*δ*) makes 1−*δ* small, favoring FD, as does any increase in fecundity with age (*α*).

Equations 1–3 show that delay can be favored even if it does not increase fecundity, but there are also cases where delay will increase fecundity, as modelled in Equations 5–8. In such cases, can we partition the benefits of delay into those that depend on increased fecundity, versus those that result solely from increased representation in a future population?

The facultative-delay genotype (FD) postpones reproduction until its second summer if environmental cues predict a population decrease before then. If the second year is also unfavorable, then this delay has no effect on FD's individual fecundity, because its reproductive success in either bad year is equivalent. However, if the next year is favorable, delay results in an increase in FD's reproductive success. Therefore, to determine how much of the increase in FD's fitness is due to increases in its individual reproductive success, we subtract the relative fitness of FD 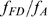 during BG years (when delay increases FD's fecundity) from its relative fitness during BB years (when delay does not increase FD's fecundity):
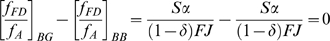
(9)Surprisingly, perhaps, increased fecundity from delay makes no contribution to relative fitness. The benefit of increased individual reproductive success by FD is exactly balanced by increases in the reproductive success of the next generation of genotype A, which never delays reproduction. Thus, under our assumptions, the fitness benefit of reproductive delay is entirely due to increased proportional representation of the alleles causing delay, not an increase in reproductive success from waiting for better conditions.

**Figure 1:**
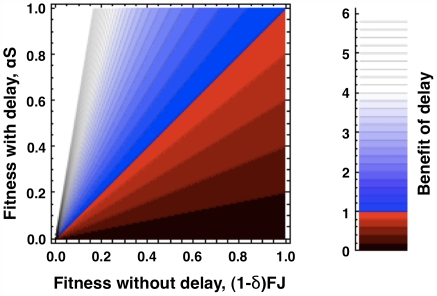
Fitness consequences of reproductive delay during population decline. An initially rare genotype (FD) that facultatively delays reproduction during a bad year is evolutionarily favored (blue shading) when *αS*, the increased fecundity of older individuals, reduced by the chance of dying before second-year reproduction, is greater than the overall change (decrease) in population size. This change, *(1−δ)FJ*, is also the reproductive success of the initially predominant annual genotype (A). This fitness landscape does not change regardless of whether conditions are better next year or not, demonstrating that the fitness benefit of delay is due, not to an increase in fecundity with improved conditions, but rather to an increase in the proportional representation of FD in a shrinking population.

The fitness consequences of variation in key life-history parameters are shown in Figure 1. Although age-linked increased reproduction by second year FDs (*α*) favors delay (Eqn. 8 and Fig. 1), *α*>1 is not required for selection to favor FD. Even if *α*<1 so that aging reduces reproductive success, reproductive delay can still be favored, so long as 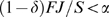. If stress is correlated with population decline (but not a perfect predictor), some bet-hedging in reproductive delay [11], [14] may be evolutionarily favored.

## Discussion

Although some of our detailed predictions might depend on our specific assumptions, such as semelparity, tradeoffs between current and future reproduction are probably universal even in iteroparous species like humans. As with Williams' antagonistic pleiotropy hypothesis [24], we accept multiple mechanisms for tradeoffs between reproduction and survival. Reproduction can increase immediate or subsequent mortality due to harmful male-female interactions during mating [28], [29], fights over mates or breeding territory, sexually-transmitted disease, or an increased risk of predation, in addition to the direct metabolic costs of reproduction and care of young. Body size, metabolic rate, blood pressure, and hormone levels that are optimal for reproduction are often not optimal in terms of longevity. For example, increased fecundity at the expense of longevity has been ascribed to high levels of insulin and insulin-like growth factor in taxa as diverse as yeast, *Drosophila*, *C. elegans*, and mammals [15]. Therefore, physiological or behavorial responses that delay reproduction will often increase longevity as a side-effect.

Our evolutionary model makes several predictions that could be tested in various species [30]. First, cues that predict overall population decline may trigger different behaviors or physiological states than cues specific to an individual's own likely survival and reproduction. For example, low current food intake but high fat reserves might predict, respectively, an overall population decrease (*FJ*<1) but a greater individual chance (*S′*) of surviving to reproduce in a subsequent year. In this case, two seemingly conflicting indicators both favor delaying reproduction, which will often increase longevity. The observation that food odors can partially reverse the effects of dietary restriction on longevity [1], [16] is consistent with this hypothesis, if food odors predict the availability of resources linked to overall population growth.

Second, cues unrelated to food supply that reliably predicted population decline over the evolutionary history of a species should also tilt the balance towards later reproduction, often increasing longevity. Facultative delayed reproduction in response to other cues of impending population decline, such as population density, weather, predation or territorial conflict may be common. These responses could be linked to undiscovered physiological mechanisms with possible medical applications. For example, the nematode *C. elegans* delays reproduction and extends lifespan by forming the relatively inactive dauer stage. Recovery from this state is stimulated by food but repressed by a pheromone that indicates high population density [5]. The interaction of these signals acts in the direction predicted by our hypothesis, favoring earlier reproduction when overall population is likely to increase.

Third, directly harmful effects of environmental factors may sometimes be outweighed by indirect health benefits linked to the reduced fecundity they trigger. For example, moderate consumption of foods containing plant defensive toxins (e.g., glucosinolates, catechins, curcumin, resveratrol) can induce similar changes in gene regulation as dietary restriction [9], delaying reproduction and increasing longevity [31]. The xenohormesis hypothesis explains this as a form of interspecific eavesdropping: organisms have evolved to respond to stress-linked phytochemicals as an early warning of environmental degradation [21]. Indeed, many of these plant defensive compounds are synthesized in response to stresses that slow plant growth, and their ingestion may thus predict a reduction in food availability, starvation, and a decline in overall population size. Alternatively, ingestion of plants with high constituitive levels of defensive toxins may result from a lack of less-toxic preferred foods. Under this “famine food” hypothesis, ingestion of these toxins, as well as spoilage indicators such as fermentation by-products, predicts starvation and short-term population decline, favoring physiological changes that delay reproduction but improve short-term health.

Those focused on human health are naturally more interested in proximate mechanisms of aging than in ultimate evolutionary explanations. With respect to the former, we agree that it may be necessary to “generalize with caution” [19]. However, our evolutionary argument is sufficiently general that it should apply to all species and to a wide variety of environmental cues.

## Methods

Figure 1 was generated using Mathematica 7.0.
